# Differential T-cell responses in dogs with meningoencephalomyelitis of unknown origin compared to healthy controls

**DOI:** 10.3389/fvets.2022.925770

**Published:** 2022-08-04

**Authors:** Renee Barber, James Barber

**Affiliations:** ^1^Department of Small Animal Medicine and Surgery, College of Veterinary Medicine, University of Georgia, Athens, GA, United States; ^2^Department of Infectious Diseases, College of Veterinary Medicine, University of Georgia, Athens, GA, United States

**Keywords:** meningoencephalomyelitis of unknown origin (MUO), T-helper cell, interferon-gamma, interleukin 17, flow cytometry

## Abstract

Meningoencephalomyelitis of unknown origin (MUO) is a common disorder in dogs that results in mononuclear inflammation in the brain and/or spinal cord. MUO is presumed to be autoimmune but specific immunological aberrations have not been identified. This exploratory study aimed to evaluate T cell production of two cytokines commonly implicated in autoimmune disease, interferon-gamma (IFNg) and interleukin-17 (IL17). Peripheral blood mononuclear cells were obtained from 12 dogs with MUO and 10 healthy controls, stimulated to activate intracellular signaling pathways, and stained with a cluster of differentiation 4 (CD4), cluster of differentiation eight (CD8), IFNg, and IL17 antibodies prior to analysis by flow cytometry. Mean differences in absolute cell numbers are represented as MUO cases minus healthy controls, and 95% Cis are reported. Overall IFNg-producing lymphocytes (mean difference = 241.8 cells/ul, 95% CI = 65.6 to 418.1) and CD4+ IFNg-producing T-cells (mean difference = 188.4, 95% CI = 77.3 to 299.5) were fewer in MUO cases. Additionally, CD4+ IL17-producing T-cells were greater in MUO cases (mean difference −34.9, 95% CI = −50.54 to −19.17) and CD8+ IL17-producing T-cells were fewer in MUO cases (mean difference = 73.5, 95% CI = 6.8 to 140.1). These results support that immunological changes can be identified in peripheral blood cells of dogs with MUO and suggest that T-helper type 17 (Th17) cells may play a role in pathogenesis.

## Introduction

Meningoencephalomyelitis of unknown origin (MUO) is a presumptive autoimmune disease affecting the central nervous system (CNS) of dogs. It is overrepresented in young to middle-aged toy and small breed dogs and after histological evaluation of tissue can be divided into three variants: granulomatous meningoencephalomyelitis (GME), necrotizing meningoencephalitis (NME), and necrotizing leukoencephalitis (NLE). Affected dogs have progressive neurological deficits and unpredictable, often temporary responses to standard immunosuppressive therapy. The etiopathogenesis of MUO is poorly understood, which has precluded the development of accurate antemortem diagnostic tests and consistently effective treatments.

Evaluation of brain tissue from MUO-affected dogs revealed elevated interferon-gamma (IFNg) and interleukin-17 (IL17) mRNA levels and protein (1), suggesting Th1 and Th17 responses may contribute to inflammation in cases of MUO. Th1 and Th17 responses are known to be important in many autoimmune diseases. In people, Th1 and Th17 have been shown to play a key role in the pathogenesis of numerous autoimmune diseases, including ankylosing spondylitis, rheumatoid arthritis, multiple sclerosis, systemic lupus erythematosus, and inflammatory bowel disease (2–5). Th17 responses have also been implicated in several canine diseases, including immune-mediated hemolytic anemia and steroid-responsive meningitis arteritis (6, 7).

This exploratory study was designed to evaluate T-cell cytokine production of IFNg and IL17 in the peripheral blood of dogs with MUO compared to healthy controls. Specifically, the authors expected to identify more clusters of differentiation 4 (CD4)+ IFNg+ and CD4+ IL17+ T-cells in MUO cases than controls.

## Materials and methods

### Case identification

This prospective study was approved by the University of Georgia Clinical Research Committee and performed with the informed consent of the owners. From 2018 to 2022, 12 dogs with MUO and 10 healthy dogs were enrolled in the study. Diagnostic inclusion criteria for MUO were based on a previous study with minor modifications (8): (i) evaluation by a neurology resident or board-certified neurologist; (ii) patient age 6 months to 12 years; (iii) focal or multifocal neuroanatomical lesion localization; (iv) focal or multifocal T2-weighted hyperintensities on magnetic resonance imaging consistent with inflammation; (v) cerebrospinal fluid (CSF) pleocytosis (> five total nucleated cells per microliter with <4,000 red blood cells per microliter) with >50% mononuclear cells; and (vi) where possible, exclusion of infectious diseases by negative serology for *Toxoplasma gondii, Neospora caninum*, and *Cryptococcus spp*. If serology for infectious diseases was not obtained, patients had to have a successful outcome (no relapse or death) for a minimum of 12 months while receiving immunosuppressive therapy or confirmation of GME, NME, or NLE by histopathology. Patients were excluded if they had received immunomodulatory therapy within the 12 weeks preceding sample acquisition. Where possible, necropsy results were recorded, but were infrequently available because the majority of cases were alive at the time of writing this manuscript.

### Flow cytometry

Cell isolation and flow cytometry were performed as previously described (9–13). Specifically, peripheral blood mononuclear cells (PBMCs) were isolated from sodium-heparinized peripheral blood using density gradient centrifugation with Ficoll-Hypaque (Histopaque^®^-1077, Sigma-Aldrich). PBMCs at the interface were collected and washed two times with phosphate-buffered saline (PBS). Cells were adjusted to a concentration of 1 x 10^6^ cells/ml and suspended in RPMI 1,640 with GlutaMAX^TM^ and HEPES (Thermo Fisher Scientific) supplemented with 10% heat-inactivated fetal bovine serum (Thermo Fisher Scientific). Cells were stimulated *ex vivo* for 4 h at 37° with and without 25 ng/ml Phorbol 12-Myristate 13-Acetate (PMA, Sigma Aldrich) and 500 ng/ml ionomycin (Sigma-Aldrich) in the presence of a 1:1,000 dilution of GolgiPlug^TM^ Protein Transport Inhibitor (containing Brefeldin A) (Thermo Fischer Scientific) (14). Cells were then washed in serum-free PBS medium containing GolgiPlug and stained with the LIVE DEAD^TM^ Fixable Aqua Dead Cell Stain Kit (Invitrogen). Cells were washed with Flow Staining Buffer (eBioscience) containing GolgiPlug and stained with anti-canine CD4-Pac Blue (clone YKIX302.9, Bio-Rad) and anti-canine CD8-AF700 (clone YCATE55.9, Bio-Rad) antibodies prior to being fixed and permeabilized (Foxp3 Transcription Factor Staining Buffer Set, eBiosience). Anti-bovine IFNg-RPE (clone CC302, Bio-Rad), and anti-human IL17/IL-17A (R&D Systems) were used for intracellular staining. The anti-IL17 antibody was conjugated using the Zenon^TM^ Alexa Fluor^TM^ 647 Goat IgG Labeling Kit (Thermo Fisher Scientific) per the manufacturer's instructions. Samples were analyzed on an LSR-II (BD Bioscience) or a Quanteon (Agilent) flow cytometer analyzer. Analysis gates were set on lymphocytes according to forward and side scatter properties with a stopping gate of 30,000 single, viable CD4+, and/or CD8+ cells. Flow cytometry (FC) data were analyzed using FACSDiva (BD Biosciences) or NovoExpress (Agilent) software. Minimum thresholds of positivity were determined by employing fluorescence-minus-one controls and, for the IFNg and IL17, verified with unstimulated (PMA and ionomycin negative) biologic controls. Relative percent FC data were translated into absolute cell counts as a product of percent IFNg or IL17 of Th or Tc, percent T-cell subset of lymphocyte gate, and absolute lymphocyte cell count data from a current clinical CBC test (<24 h prior).

### Statistical analysis

Absolute cell numbers for the following categories were analyzed between MUO cases and healthy controls by the unpaired *t*-test with the Welch's correction using the GraphPad Prism version 9: IFN-g+ lymphocytes, CD4+ IFNg+ T-cells, CD8+ IFNg+ T-cells, IL17+ lymphocytes, CD4+ IL17+ T-cells, and CD8+ IL17+ T-cells. Q–Q plots of the residuals were examined to confirm the assumption of approximate normality. There was non-homogeneous variance with the diseased group having higher variability than the healthy control group generally, so the Welch's *t*-tests were used. Mean differences in absolute cell numbers are represented as MUO cases minus healthy controls. Ninety-five percentage of CIs are reported.

## Results

### Case information

A total of 12 MUO cases were evaluated, including three Maltese, three mixed breeds, and one each of Pomeranian, Boston terrier, Jack Russell terrier, Shih Tzu, Chihuahua, and Yorkshire terrier.

The mean age was 6.9 years (range 2–12 years). Among the animals, nine were spayed females and four were neutered males. Necropsy results were available for one case with a diagnosis of GME.

A total of 10 healthy controls were evaluated, including three mixed breeds, two Maltese, two poodles, two Yorkshire terriers, and one Chihuahua. The mean age was 6.6 years (range 2–12 years). Among the animals, six were spayed females and four were neutered males.

### Flow cytometry

To identify CD4+ and CD8+ T-cells producing IFNg and IL17, we performed a flow cytometric analysis of PBMCs stimulated with PMA and ionomycin (Supplementary Figure S1).

**Figure 1:**
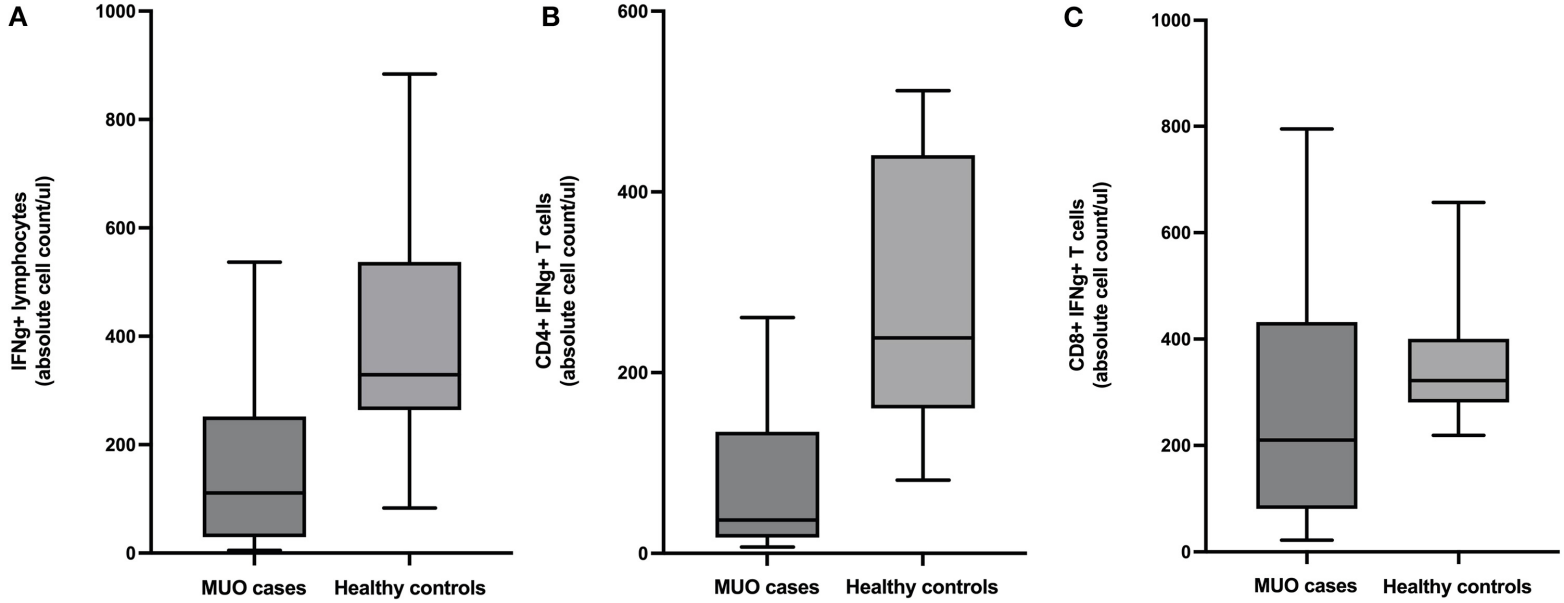
Box and whiskers plots showing absolute cell counts of **(A)** IFNg+ lymphocytes, **(B)** CD4+ IFNg+ T-cells, and **(C)** CD8+ IFNg+ T-cells in cases of meningoencephalomyelitis of unknown origin (MUO) versus healthy controls.

There were fewer lymphocytes producing IFNg in MUO cases than healthy controls (mean difference = 241.8, 95% CI = 65.6 to 418.1, *p* = 0.01). Additional analysis of lymphocyte subpopulations revealed there were fewer CD4+ IFNg+ T-cells in MUO cases compared to healthy controls (mean difference =188.4, 95% CI = 77.3 to 299.5, *p* = 0.003) and fewer CD8+ IFNg+ T-cells in MUO cases compared to healthy controls (mean difference = 91.9, 95% CI = −73.8 to 257.6, *p* = 0.26) (Figure 1).

**Figure 2:**
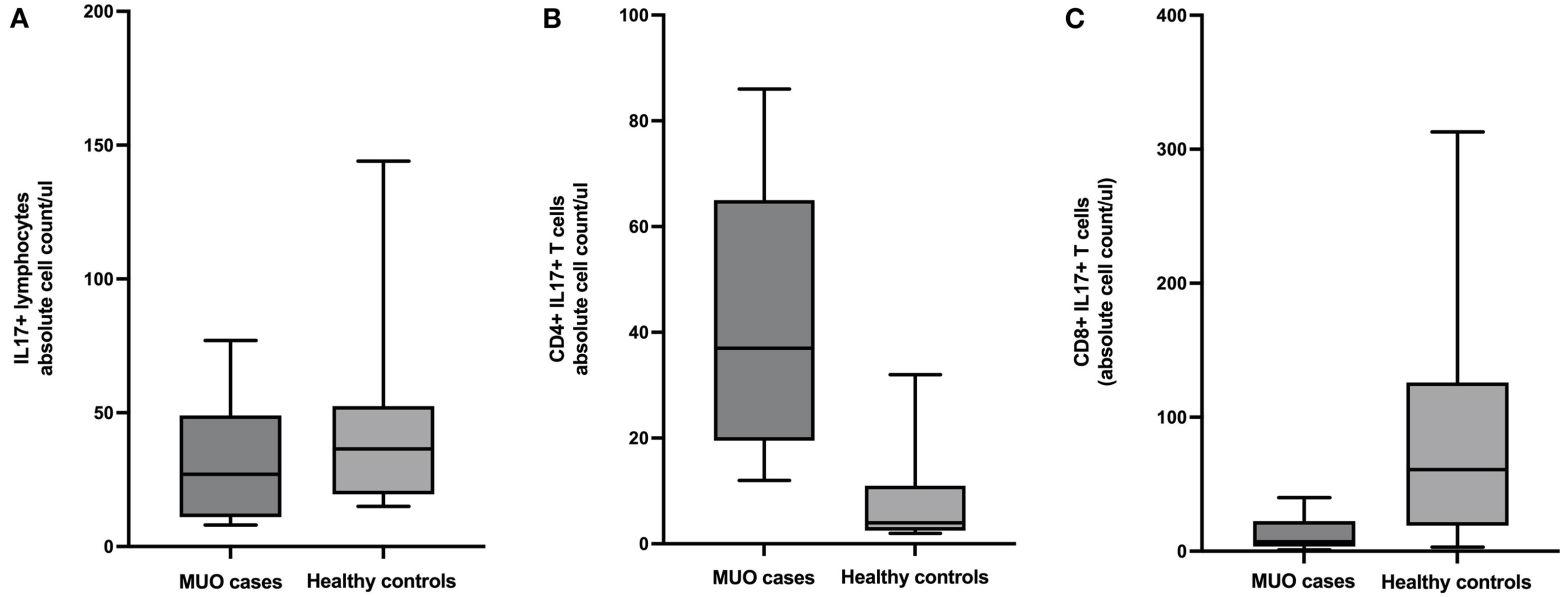
Box and whiskers plots showing absolute cell counts of **(A)** IL17+ lymphocytes, **(B)** CD4+ IL17+ T-cells, and **(C)** CD8+ IL17+ T-cells in cases of meningoencephalomyelitis of unknown origin (MUO) versus healthy controls.

There were fewer lymphocytes producing IL17 in MUO cases compared to controls (mean difference =13.78, 95% CI = −15.3 to 42.9, *p* = 0.33). Additional analysis of lymphocyte subpopulations revealed there were more CD4+ IL17+ T-cells in MUO cases compared to healthy controls (mean difference = −34.9, 95% CI = −50.54 to −19.17, *p* = 0.0002) and fewer CD8+ IL17+ T-cells in MUO cases vs. healthy controls (mean difference =73.5, 95% CI = 6.8 to 140.1, *p* = 0.03) (Figure 2).

## Discussion

Analysis of lymphocyte populations in MUO cases vs. healthy controls identified differences in IFNg- and IL17-producing cells. There were fewer IFNg-producing lymphocytes, specifically characterized by fewer CD4+ IFNg+ T-cells, in MUO compared to controls. Additionally, there were more CD4+ IL17-producing T-cells but fewer CD8+ IL17-producing T-cells in MUO compared to controls. The results of this exploratory study support that immunological differences can be identified in the peripheral blood of dogs with MUO and yield some insight into pathogenesis, suggesting IL17 and Th17 responses may play a role in disease pathogenesis.

There are only a small number of studies looking at specific components of the immune system in dogs with MUO. Several groups have used immunohistochemistry to look at B and T-cells in GME lesions with differing results as to the predominant cell type (15–18). Other groups have looked at various cytokines and chemokines in brain tissue and CSF (19). Specifically, evaluation of mRNA and protein expression levels in brain tissue from a small number of cases revealed elevated IFNg in NME and elevated IL17 in GME (1). To the author's knowledge, no studies looking at peripheral components of the immune system in MUO have been published. Although it is hard to compare the evaluation of brain tissue with peripheral blood, the findings presented here are consistent with the identification of elevated IL17 in brain tissue of GME. However, in that study, IL17 was more commonly associated with macrophages than T-cells (1) and in the study presented here, histological evaluation to determine the MUO subtype could not be performed for all cases.

There are several limitations to this study. This was an exploratory study so only small numbers of cases and controls were utilized, which can increase the margin for error and, and additionally, precluded correlation of results with clinical findings. Where possible, controls were age and breed matched but this was not always possible. Also, the diagnosis was not confirmed by histopathology in the majority of dogs.

Ultimately, this exploratory study suggests that immunological differences can be identified in peripheral blood from dogs with MUO and supports that IL17-producing T-cells may play a role in disease pathogenesis. These results should be validated with a larger sample size prior to additional research to determine the biological and clinical significance of these changes. Ultimately, components of Th17 responses may provide useful biomarkers and could inform the development of targeted immunotherapies.

## Data availability statement

The original contributions presented in the study are included in the article/Supplementary material, further inquiries can be directed to the corresponding author/s.

## Ethics statement

The animal study was reviewed and approved by Clinical Research Committee at the University of Georgia College of Veterinary Medicine. Written informed consent was obtained from the owners for the participation of their animals in this study.

## Author contributions

RB contributed to study design, case identification, sample acquisition and processing, and manuscript preparation. JB contributed to study design, sample processing, data analysis, and manuscript preparation. Both authors contributed to the article and approved the submitted version.

## Funding

Funding was provided by the American Kennel Club Canine Health Foundation (grant # 2452).

## Conflict of interest

The authors declare that the research was conducted in the absence of any commercial or financial relationships that could be construed as a potential conflict of interest.

The handling editor AT, declared a past co-authorship with the author, RB.

## Publisher's note

All claims expressed in this article are solely those of the authors and do not necessarily represent those of their affiliated organizations, or those of the publisher, the editors and the reviewers. Any product that may be evaluated in this article, or claim that may be made by its manufacturer, is not guaranteed or endorsed by the publisher.
