# Supplementary material for: Differential T-cell responses in dogs with meningoencephalomyelitis of unknown origin compared to healthy controls
Source: Front Vet Sci. 2022 Aug 4;9:925770. doi: 10.3389/fvets.2022.925770 (PMC9386037; doi:10.3389/fvets.2022.925770)
Supplement: Supplementary file 1 [file Data_Sheet_1.PDF]

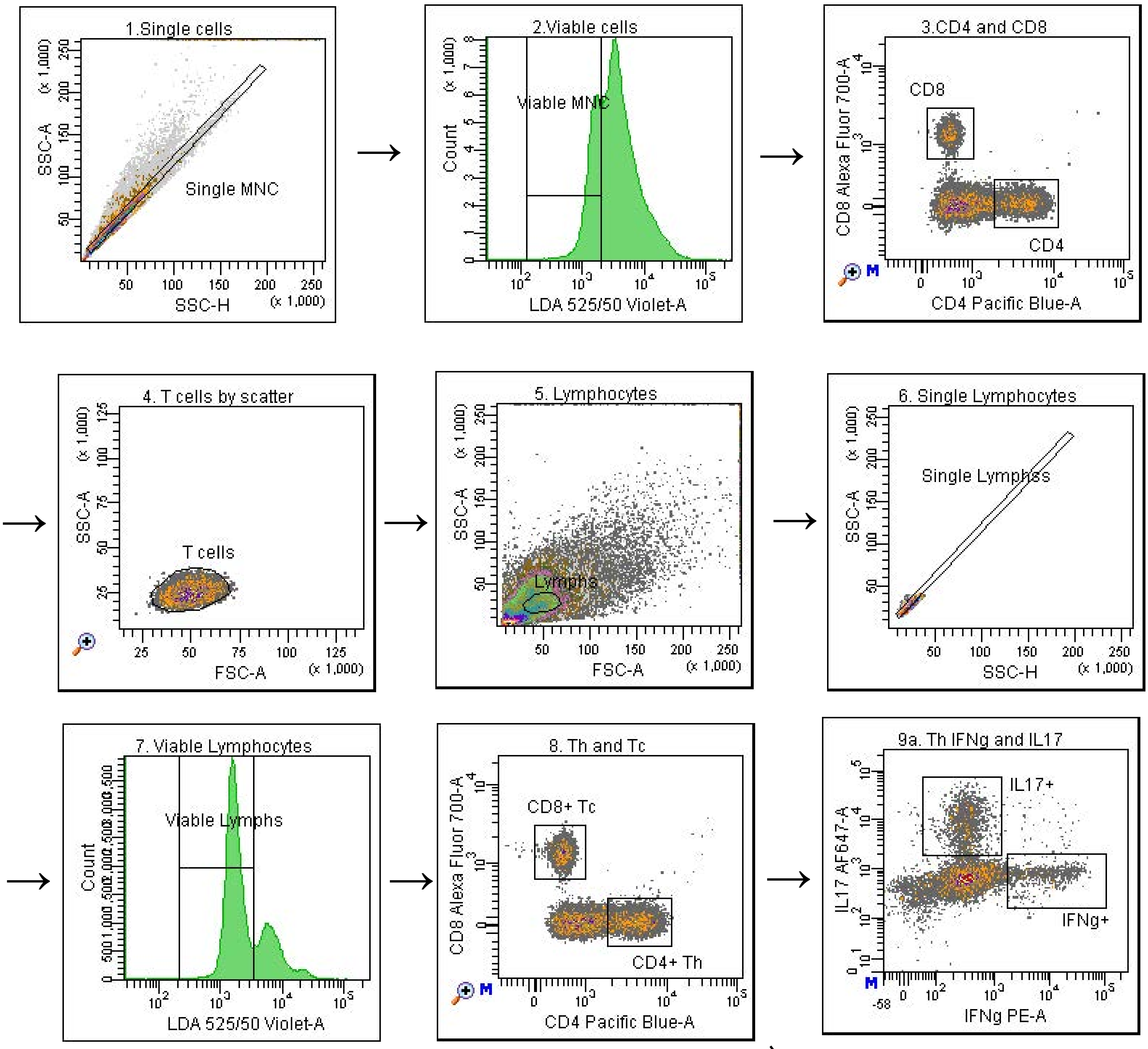

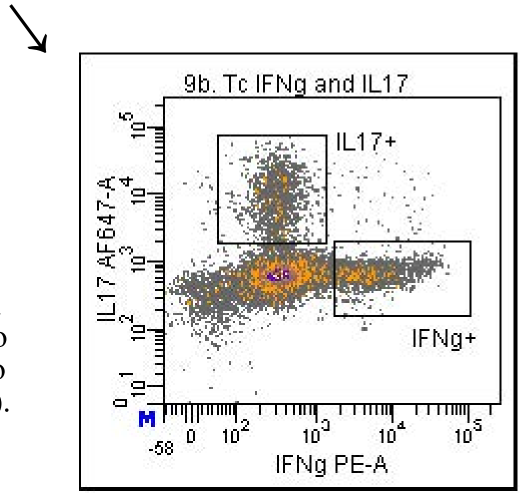

FIGURE S1. Flow cytometric analysis workflow. All cells were subject to single-cell discrimination using pulse-width (1) followed by identification of viable cells with exclusion of a fixable amine-reactive dye (2). CD4- and CD8-positive cells (3) were back-gated on diffracted (FSC) and refracted (SSC) light scatter to determine the best lymphocyte scatter gate to use for subsequent T-cell subset analysis (4). Light-scatter gated lymphocytes (5) were then re-subjected to singlet (6) and viability (7) gating. Viable, single lymphocytes were grouped into T-helper (CD4+) or T-cytotoxic (CD8+) T-lymphocyte subsets, or other cells (8). Relative percents of IFNg- and IL17-positive cells were assessed for T-helper (9a) and T-cytotoxic (9b) cells.
